# Evaluation of a Commercial Electronic Nose Based on Carbon Nanotube Chemiresistors

**DOI:** 10.3390/s23115302

**Published:** 2023-06-02

**Authors:** Ruud Peters, Niels Beijer, Bauke van ‘t Hul, Brigitte Bruijns, Sandra Munniks, Jaap Knotter

**Affiliations:** 1 Lectorate Technologies for Criminal Investigations, Saxion University of Applied Sciences, Handelskade 75, 7417 DH Deventer, The Netherlands; 2 Academy of Applied Biosciences and Chemistry, HAN University of Applied Sciences, Laan van Scheut 2, 6525 EM Nijmegen, The Netherlands; 3 Wageningen Food Safety Research, Wageningen University and Research, Akkermaalsbos 2, 6708 WB Wageningen, The Netherlands; 4 Dutch Police Academy, Arnhemseweg 348, 7334 AC Apeldoorn, The Netherlands

**Keywords:** electronic nose, chemiresistors, carbon nanotubes, VOCs

## Abstract

Recently a hand-held, carbon-nanotube-based electronic nose became available on the market. Such an electronic nose could be interesting for applications in the food industry, health monitoring, environmental monitoring, and security services. However, not much is known about the performance of such an electronic nose. In a series of measurements, the instrument was exposed to low ppm vapor concentrations of four volatile organic compounds with different scent profiles and polarities. Detection limits, linearity of response, repeatability, reproducibility, and scent patterns were determined. The results indicate detection limits in the range of 0.1–0.5 ppm and a linear signal response in the range of 0.5–8.0 ppm. The repeatability of the scent patterns at compound concentrations of 2 ppm allowed the identification of the tested volatiles based on their scent pattern. However, the reproducibility was not sufficient, since different scent profiles were produced on different measurement days. In addition, it was noted that the response of the instrument diminished over time (over several months) possibly by sensor poisoning. The latter two aspects limit the use of the current instrument and make future improvements necessary.

## 1. Introduction

An electronic nose is a measuring instrument that mimics the mammalian olfactory system and gives fingerprint information of mixed gases or odors as output. In general, an electronic nose is composed of two parts: a gas sensing system consisting of chemical sensors and an information processing system with pattern recognition software. It has been more than 30 years since the electronic nose concept was introduced in the 1980s [1,2]. Since then, electronic noses have evolved from being large in size, expensive, and power consuming instruments to portable, low cost devices with low power consumption. Electronic noses have been applied in many fields such as food analysis [3,4,5], health [6,7], environmental monitoring [8,9], and security [10,11]. For more information about electronic noses, two recently published review papers that describe the development and applications of the electronic nose can be consulted [12,13].

In an electronic nose, chemical sensors are used for detecting chemicals in the environment, often air. Basically, these sensors convert chemical information into analytical signals. The main objective of an electronic nose is to detect a scent or odor, and since these are generally composed of multiple chemicals, electronic noses should sense more than one chemical. In practice this is achieved by combining multiple distinct sensors in a sensor array taking into account the chemicals of interest. During the last three decades, different sensor types have been utilized in the electronic nose technology, and these are briefly summarized here. Metal-oxide sensors (MOS) are the most used sensor types in electronic noses because of their suitability for a wide range of gases, fast response, small size, and ease of use [14]. Because oxide surface reactions are slow at lower temperatures, these sensors operate at high temperatures and, therefore, require high power consumption. The detection range typically varies from 5 to 500 ppm [14]. Other types of gas sensors used in electronic noses are electrochemical sensors. In these sensors, electrochemical oxidation or reduction of molecules occurs on a catalytic electrode surface. The dynamic concentration range varies from 10 to 1000 ppm [4]. Conducting polymer (CP) sensors are also used in electronic noses [15]. When a change occurs in the polymer material of a CP sensor due to an interaction with an analyte, the resistance in the sensor changes which leads to the detection of molecules. A typical dynamic concentration range of this type of sensor is 0.1–100 ppm and are used due to their low cost, fast response, and resistance to sensor poisoning. Quartz crystal microbalance (QCM) sensors have also been used as electronic nose components in several applications [16]. A selective barrier on the crystal surface takes in the analytes from the environment, which then increases the total mass. Subsequently, the crystal frequency decreases due to the mass change on the crystal surface. While differences of 1 ng in mass can be detected, the signal-to-noise ratio is rather poor. Finally, surface acoustic wave (SAW) sensors are frequently used in electronic nose applications because of their small size, sensitivity, low cost, and response to nearly all gases [17]. SAW sensors operate in a similar way as QCM sensors, but they are less sensitive with a detection limit of about 10 ng in mass.

More recently, carbon nanotubes (CNTs) have been used in electronic noses [18,19]. Different from MOS sensors, CNTs do not require high temperatures and have, therefore, low power consumption which is a positive feature. CNTs are good candidates for gas sensing because they can translate changes in their local chemical environment into electrical signals to sense chemical species. Their application can be attributed to their high surface area to volume ratio, dimensionality in the nanoscale, and resistance that is dependent on their local chemical potential. The nanotubes are often functionalized with materials such as metal oxides, noble metal nanoparticles, and polymers to improve sensitivity and selectivity [20,21,22]. The nanotube chemiresistors also need a corresponding measurement device to measure the changes in their electrical properties to be considered a fully functioning sensor.

This paper describes the performance of a portable, carbon-nanotube-based, electronic nose that recently became available on the market. While the number of commercial devices increases there is the necessity of independent evaluations of their features. The question that needs to be answered is whether this electronic nose is a “gadget” or whether it can be a serious measuring instrument useful for the food industry, health monitoring, and environmental monitoring. To do so, low ppm vapor concentrations of four volatile organic compounds were generated to see whether the instrument can detect these vapors and determine a scent pattern that allows the identification of these four vapors. Furthermore, performance characteristics such as detection limits, repeatability, and reproducibility of signal height and scent pattern were determined.

## 2. Materials and Methods

**Figure 1:**
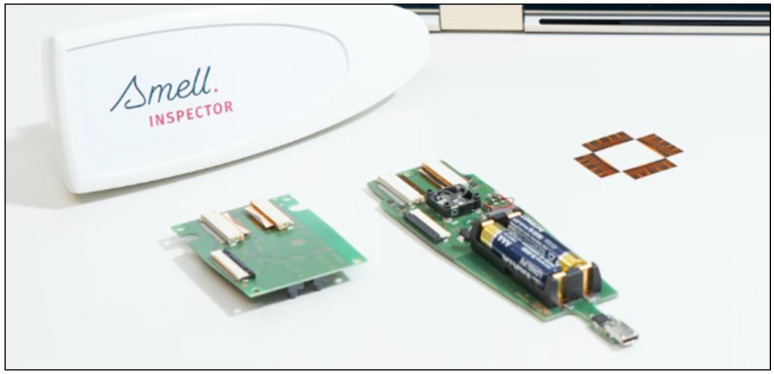
The Smell Inspector, exterior and interior. At the top left, the housing of the instrument is shown, and directly below it, the two printed circuit boards inside the instrument. The polymer-coated four sensor chips can be recognized as the white rectangles at the top of the two printed circuit boards. On the right-hand side, the four sensor chips without the polymer coating are shown. Finally, at the right-hand bottom, the USB connector is visible which connects the instrument to a laptop computer equipped with the Smell Annotator software.

The electronic nose used in this study is the Smell Inspector from Smart Nanotubes [23], see Figure 1. The manufacturer claims that their electronic nose is based on the work of Panes-Ruiz et al. [24].

The Smell Inspector contains four chips, each containing 16 independent gas sensors, as well as humidity and temperature sensors. According to the manufacturer, the sensor elements contain fine-tuned carbon nanomaterials with a size of less than 1 sqmm each, requiring only 1 µW of power supply. The read-out electronics, which are incorporated in the Smell Inspector, sample the multiple sensors every 1.8 s by recording the sensor’s electrical resistance. Each sensor responds differently when exposed to a single gas, resulting in a unique pattern of electric signals from the sensor array, comparable with a digital fingerprint. Although individual sensors are usually not highly selective, their combined signals allow the characterization of samples in their entirety. The flow rate of air/gas through the Smell Inspector housing and over the sensor chips can be adjusted by selecting different fan speeds and ranges from about 5 to 25 mL/min. The Smell Inspector is connected to a PC using a USB connection, and Smell Annotator software is used to detect, annotate, and digitize odors.

After switching on the instrument, it is left for 30 min to equilibrate and stabilize the output signal. Measurement is started using the Smell Annotator software, and the response of the electronic nose to selected volatile organic compounds (VOCs) was investigated with a static gas generation system at room temperature. Typically, 10 µL of the liquid VOC was allowed to vaporize in a 5 L glass bottle filled with purified air and placed on a hot plate with a temperature of 40 °C to add vaporization. After full evaporation and conditioning for 15 min, a gaseous subsample in the order of mL is collected from the bottle and injected into a Tedlar bag (SKC, Dorset, UK) of 0.5, 1, 2, or 5 L and filled with purified air. As an example, for toluene the addition of 10 µL to the 5 L glass bottle after evaporation results in a gaseous toluene concentration of 1730 mg/m^3^, which translates to 454 ppm. Next, a gaseous subsample of 9 mL is collected with a gas-tight injection syringe and injected into a 2 L Tedlar bag filled with purified air resulting in a final concentration of 2 ppm. The relative humidity of the test gas mixture was 40%. The outlet of the Tedlar bag is directly connected to the inlet of the Smell Inspector with a piece of tubing. Individual gas concentrations of methylamine, acetonitrile, ethyl acetate, and toluene at 2 ppm were prepared. These compounds were chosen because of their difference in odor and molecular polarity (the relative polarities of these compounds are 0.76, 0.46, 0.23, and 0.01, respectively [25]. The electronic nose was exposed to concentrations of the single VOCs by passing the gaseous vapors over the carbon nanotube chemical sensor array using the built-in fan in the detector housing set at its maximum. The flow rate is estimated to be 25 mL/min. To obtain the sensing response to a VOC, the reference baseline was defined during the first 3 min without exposure to any VOC. Then, the detector was exposed to the VOC concentration, and the output signals were recorded for 5 min to reach a steady-state condition. The experiments were carried out in a conditioned laboratory with a temperature of 21 °C and a relative humidity of 40%.

The Smell Inspector contains four chips, each containing 16 independent gas sensors as shown in Table 1. The sensor response is read in the form of the resistance across the active layer of each sensor and hence, each measurement produces a 64-channel time series. Sensors with the same number have the same chemical functionalization and have, therefore, the same characteristics and resistance values upon exposure to VOCs. The use of such replicates (here n = 3) in a sensor array will result in higher precision and accuracy as shown by Payette et al. [26]. From the raw data file, it became clear that not all 64 sensors (4 chips × 16 sensors) deliver data or are used in data processing. In total 19 sensors, indicated in the raw data by “#functionalization 999”, are not used or reflect only the base resistance values. A schematic of the active and non-active sensors is given in Table 1.

Although the Smell Annotator software offers some possibilities to process the raw data, the export function was used to export the raw data as a CSV file which was imported into Microsoft Excel Office 360. The raw data consists of 64 sensor resistance value cycles plus temperature and humidity values. The fractional method was applied to the determined sensor resistances to calculate the sensor responses as follows:

(1) $$\%Sensor\;response=\frac{R_{g}-R_{0}}{R_{0}}\times 100\;$$

where *R*_0_ is the base resistance of the sensor under ambient conditions without exposure to any VOC, and *R_g_* is the sensor resistance after exposure to the VOC concentration. This is comparable to the method used by Zang et al. in a recent publication [27]. Next, the mean averages of the %Sensor response of each three individual sensors with the same number, and therefore characteristics (see Table 1), were calculated. This resulted in 15 features (one for each averaged sensor group) that are characteristic of the VOC that is being measured. When the 15 features are plotted in time, this results in the response curve of the measurement. The 15 features are also plotted in a radar plot to produce an image that is characteristic of the odor that is being detected. Radar plots were produced for each of the four VOCs, methylamine, acetonitrile, ethyl acetate, and toluene. Statistical analysis was performed in RStudio (Windows version R 4.2.2.) and Excel Office 360. To determine the repeatability, 2 ppm concentrations of each of the VOCs were measured seven-fold on the same day. The repeatability was expressed as the relative standard deviation in the signal height of the seven measurements. To determine the within-lab reproducibility, the repeatability measurements of the 2 ppm concentration of toluene were repeated on two more different days which were a week apart. One-way ANOVA on all measurement results was used to calculate the reproducibility. Although the Smell Inspector is not intended as a quantitative measuring instrument, linearity was determined for toluene by measuring concentrations in the range of 0.5–8 ppm. Finally, detection limits were estimated as a signal/noise ratio of 3 for each of the VOCs from the lowest concentrations that were measured and that were still visible in the time scan.

## 3. Results and Discussion

The Smell Inspector from Smart Nanotubes is one of the first handheld electronic noses that became available on the market. From the instrument itself and the marketing it is not clear whether this is intended as a serious measurement instrument or that it is a gadget for household use. Potential applications can be found in the food industry, environmental monitoring, and security services for detecting drugs and explosives, which was the motivation to test the instrument and determine its capabilities. To do so, four VOCs with very different odors and different polarities were selected, methyl amine, acetonitrile, ethyl acetate, and toluene. Methylamine is relevant for the detection of drugs since it is used in the production of 3,4-methylenedioxymethamphetamine (MDMA, also known as XTC) and amphetamines, whereas acetonitrile is used to make pharmaceuticals and perfumes. The ester ethyl acetate is a relevant model compound for the food industry, and toluene is relevant for environmental monitoring.

**Figure 2:**
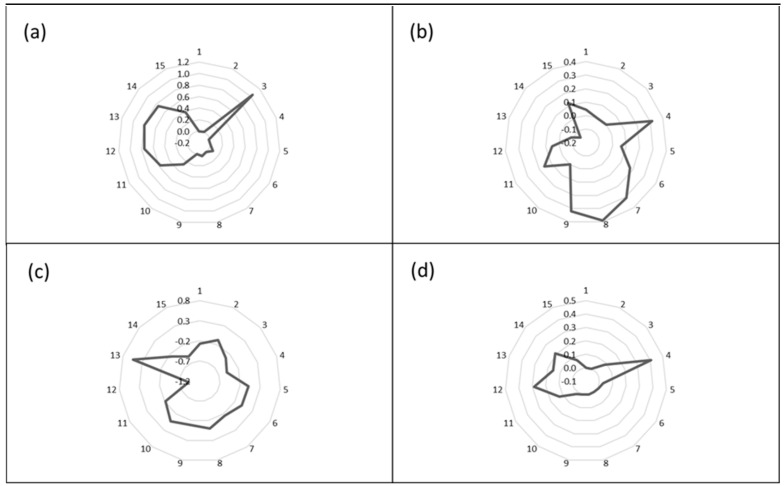
Radar plots of the four VOCs ((**a**); methylamine, (**b**); acetonitrile, (**c**); ethyl acetate, (**d**); toluene) showing clear differences in the image allowing identification of the individual VOCs.

As mentioned in the previous section the individual sensors respond differently to the exposure of a VOC, and 15 features can be derived from the raw data. The response of these 15 features can then be plotted in a radar plot effectively resulting in an image or pattern that is characteristic of the particular odor of that VOC. These odor images for methyl amine, acetonitrile, ethyl acetate, and toluene are presented in Figure 2 and were collected at single VOC exposure concentrations of 2 ppm. The Smell Annotator software can do this in analysis mode or these patterns can be calculated from the raw data which is exported to Excel from the Smell Annotator software.

The idea behind the electronic noses in general is that each scent or odor (as well as every single gas) produces a unique pattern and that a substance can be identified by this scent pattern. The Smell Annotator software, however, has no option to identify scent patterns from a database. This means that all the patterns, which correspond to different scents to be recognized later, should be recorded first. Furthermore, to use the Smell Inspector for scent identification or recognition, a machine learning (ML) model should be used. This model has to collect a sufficient number of annotated smell measurements and process them with an ML algorithm. While this option is mentioned in the Smell Annotator software, it is not yet available. In addition, a prerequisite for this to work is that the measurements with the Smell Inspector show good repeatability and reproducibility.

**Figure 3:**
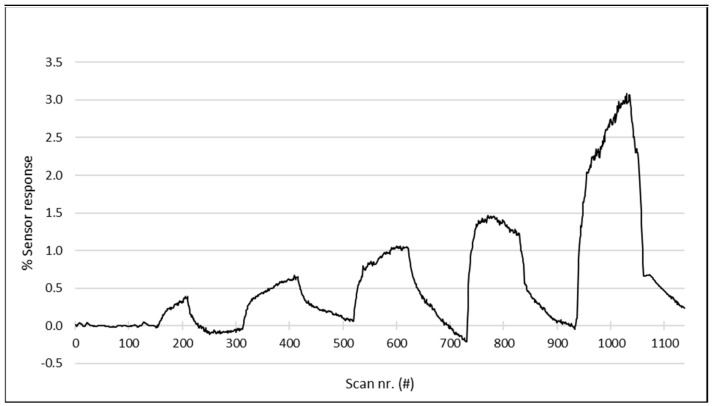
The response of the sensor with most sensitive signal for toluene vapor. The concentrations tested are from left to right, 0.5, 1.0, 2.0, 4.0, and 8.0 ppm The lowest concentration of 0.5 ppm is still visible, and the peak height (% Sensor response) seems to increase by a factor of 2 in every next peak.

**Figure 4:**
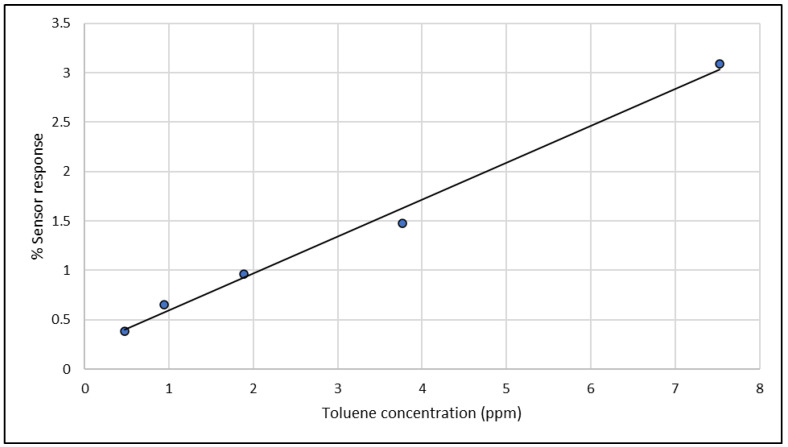
Calibration line for toluene concentrations in the range of 0.5–8.0 ppm. The correlation coefficient (R^2^ = 0.993) indicates that the response is linear in this range.

Any odor is composed of one or more volatile compounds which makes measurements to determine the performance of an instrument very complicated. Therefore, all measurements to determine the number of performance characteristics of the instrument were performed with single compounds. Since an odor can be composed of compounds present at very low concentrations (ppb range or even lower), it was important to determine the working range of the instrument. This was done by measuring toluene at different concentrations ranging from 0.5 to 8.0 ppm with concentrations increasing by a factor of 2 in every step. The results of this measurement are presented as a time series in Figure 3 which shows the response of the sensor with the most sensitive signal for toluene. As can be seen, the lowest concentration of 0.5 ppm toluene is still measurable, and the signal height increases by a factor of 2 in every next exposure. This is also clear from the calibration curve that is shown in Figure 4 and produces a correlation coefficient of 0.993, indicating a linear response for toluene concentrations in the calibration range.

From the response of the sensor for the lowest concentration of toluene (0.5 ppm), it is estimated that the detection limit of the electronic nose for toluene is 0.2 ppm. Detection limits were also estimated for the other three VOCs. For methylamine, the detection limit is estimated to be 0.1 ppm, whereas the detection limits for ethyl acetate and acetonitrile are estimated to be 0.5 ppm. Such detection limits are probably low enough to detect drug precursors in unknown powders and scent compounds in food. However, for explosives, the detection limits are probably not low enough since such compounds have generally low vapor pressures and are present in the air in much lower concentrations. From the results, it was decided to do all other measurements at a concentration of 2 ppm, well above the detection limits of the VOCs, but low enough not to overload the sensor.

**Figure 5:**
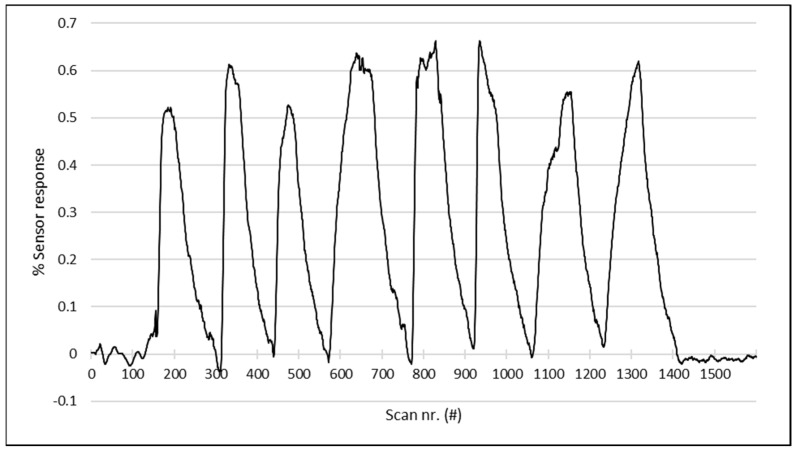
Repeatability determined by seven measurements of 2 ppm toluene on the same day.

**Figure 6:**
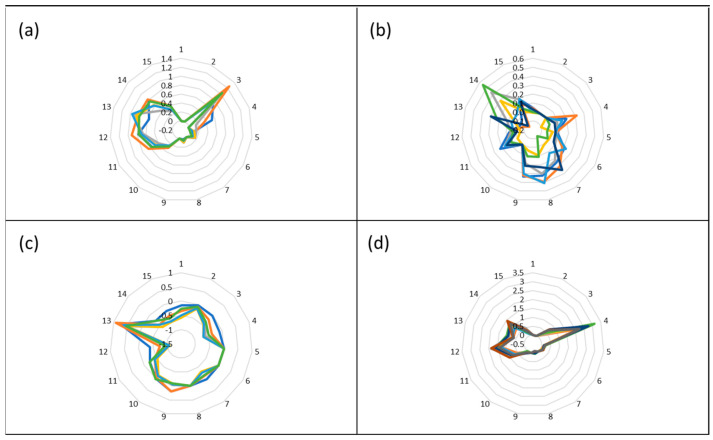
Repeatability plots of the scent pattern of the four VOCs ((**a**); methylamine, (**b**); acetonitrile, (**c**); ethyl acetate, (**d**); toluene) in the repeatability measurements.

The repeatability of the signal height was determined as the relative standard deviation in the results of seven measurements of a 2 ppm concentration of a single VOC on the same day. The time series for toluene is shown in Figure 5. Based on signal height, this resulted in repeatability for toluene of 8%. The repeatability for methyl amine, ethyl acetate, and acetonitrile was 16%, 13%, and 27%, respectively. Note that this repeatability is only for the height of the signal of the most sensitive sensor. More important, in terms of identifying a scent, is the repeatability of the scent pattern (i.e., the radar plot produced by the 15 features that are determined from the raw data). It is not easy to convert this repeatability into a number, and, therefore, the mixed radar plots of the individual repeatability measurements of the four VOCs are presented in Figure 6. While methyl amine, ethyl acetate, and toluene produce more or less the same pattern in each of the repeatability measurements, acetonitrile produces larger differences. This was already reflected by the repeatability of the signal height for acetonitrile which was also worse than for the other VOCs. This means that it will not be easy to identify acetonitrile as a scent.

**Figure 7:**
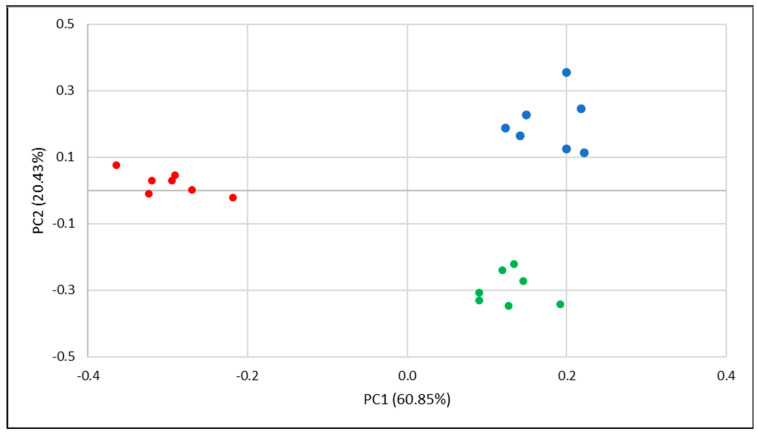
PCA plot of the repeatability data of methyl amine (green), ethyl acetate (red) and toluene (blue).

Finally, principal component analysis (PCA) was applied to the repeatability analysis data of the four VOCs. PCA reduces the complexity of high-dimensional data while preserving patterns and trends. From the PCA analysis, we can deduce (1) whether multiple measurements from the same scent can be combined in a group, (2) whether two different scents can be presented as two separated groups, and (3) how large the variance of the same scent measurement within a scent group is. Applying PCA to the repeatability data of methylamine, ethyl acetate, and toluene resulted in Figure 7. It is clear that we can recognize three groups in the PCA graph, one for each single VOC. The groups of methyl amine, ethyl acetate, and toluene form relatively confined scent groups and can very well be distinguished from the other measurements in the PCA graph. This was expected based on the similarities that were found in the scent patterns of these VOCs (Figure 6). The situation is different for acetonitrile. The differences between the scent patterns were so large that it disturbed the PCA plot to the extent that differences between the individual measurements of the other VOC were no longer visible. Therefore, the uncertainty in the identification of a new, unknown scent as acetonitrile is large.

**Figure 8:**
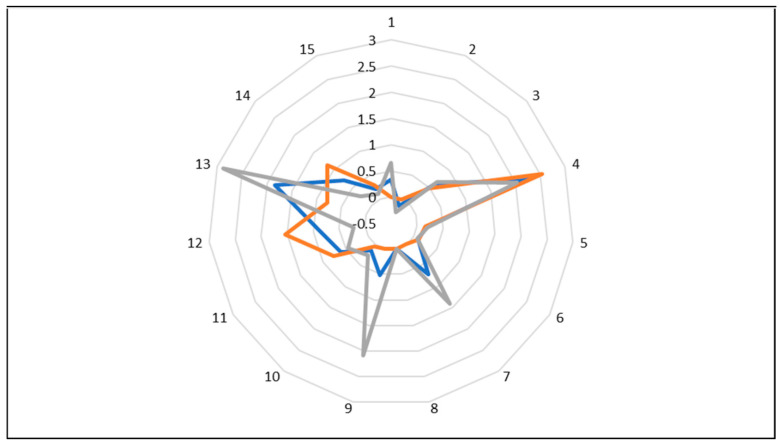
The scent pattern of toluene at 2 ppm acquired on three different days. The differences of the individual patterns illustrate the limited reproducibility.

The reproducibility of the Smell Inspector is not sufficient. In measurements performed on three different days for toluene, differences were found not only in signal height but also, more importantly, in scent pattern as presented in Figure 8. If a database for scent identification is to be prepared and used, reliable pattern determination is a prerequisite. Another potential problem is that it was noted that over a period of months, the sensitivity of the instrument was diminishing, and the response to VOC concentrations but also to concentrations of permanent gases as ammonia decreased (a low concentration of ammonia was regularly measured for quality control reasons). This may be a consequence of sensor poisoning due to irreversible adsorptions of VOCs and other gases. The bad reproducibility and decrease in sensor response are surely problems for the long-term use of the instrument.

## 4. Conclusions

In this study, a commercial electronic nose was tested for its ability to measure and differentiate scents. The results indicate that the detector can detect concentrations of VOCs with different molecular polarities as low as 0.1–0.5 ppm and show a linear response for toluene concentrations of 0.5–8.0 ppm. The repeatability of the signal height was about 8–16% for methyl amine, ethyl acetate, and toluene, whereas it was higher for acetonitrile with 27%. The repeatability measurements for methyl amine, ethyl acetate, and toluene also showed promising results for the scent patterns, and a PCA analysis showed that these VOCs can be distinguished from one another. For acetonitrile, the situation was worse indicating that this compound will not be easily identified based on its scent. The reproducibility was tested with toluene and was not sufficient since scent patterns produced on different days showed relatively large differences. Most of the results show that this commercial instrument is promising and more than a gadget. However, more research is needed into the limited reproducibility of the instrument. If this can be improved, then this commercial instrument can be a valuable tool for scent measurements in various applications.

## Figures and Tables

**Table 1:** Schematic of the active and non-active 16 sensors in each of the four chips. The numbers, e.g., 69, 41, 40, and 33 in chip 1, indicate the functionalization, and sensors with the same number have the same functionalization. The number 999 indicates that this sensor is not used or reflects only base resistance values.

	ch1	ch2	ch3	ch4	ch5	ch6	ch7	ch8	ch9	ch10	ch11	ch12	ch13	ch14	ch15	ch16
Chip1	999	999	69	69	69	41	41	41	40	40	40	33	33	33	999	999
Chip2	999	999	999	999	999	61	61	61	47	47	47	43	43	43	999	999
Chip3	999	999	90	90	90	67	67	67	53	53	53	42	42	42	999	999
Chip4	999	999	94	94	94	89	89	89	85	85	85	59	59	59	999	999

## Data Availability

The data presented in this study are available on request from the corresponding author. The data are not publicly available due to confidentiality restrictions.
